# Sense of Control and Safety Compliance in the Prevention of COVID-19: A Framework Based on Conservation of Resources Theory

**DOI:** 10.3389/fpsyg.2022.790459

**Published:** 2022-04-13

**Authors:** Pingping Li, Huaixin Zhu

**Affiliations:** ^1^School of Business, NingboTech University, Ningbo, China; ^2^College of Education, Zhejiang University, Hangzhou, China

**Keywords:** sense of control, psychological stress, safety compliance, safety participation, perceived safety regulation

## Abstract

Drawing on conservation of resources theory, this study examined how and when sense of control influence safety behavior (e.g., safety compliance and safety participation). Linear regression analysis was performed on data collected from 481 students in 58 classes at a university. The results indicated that psychological stress mediated the negative effect of sense of control on safety compliance, as well as the positive effect of sense of control on safety participation. They further showed that perceptions of stronger safety regulations heightened the positive relationship between student psychological stress and safety compliance, and buffered the negative effects of psychological stress on safety participation. These results provide a benchmark against which the effectiveness and relevance of epidemic prevention and control in higher education institutions can be assessed.

## Introduction

Noncompliance with safety policies and passively participation in safety management have been commonplace during efforts to prevent and control outbreaks of COVID-19 and its continued spread, thus adding to the risks it poses to society. For example, some people do not accept neighborhood management rules, or they advocate the violation of epidemic control policies. In colleges and universities, students can be ignorant of epidemic safety control policies and fail to actively participate in safety management (e.g., by helping their roommates report lower body temperatures and not wearing masks at school). Coupled with the crowdedness and relatively high population densities of higher education environments, these behaviors may result in large groups becoming infected in college and university communities. Therefore, the higher education environment has emerged as a key research target in the investigation of COVID-19 prevention and control (Cheng et al., 2020; Wang et al., 2020).

Given the importance of safety-related behaviors, including safety compliance (i.e., behaviors that individuals need to carry out to maintain safety; Neal and Griffin, 2006) and participation (i.e., behaviors that do not directly benefit an individual’s personal safety but do help improve conditions that support safety; Neal and Griffin, 2006), studies have thoroughly explored the antecedents of these behaviors (e.g., Griffin and Neal, 2000; Neal and Griffin, 2006; Griffin and Hu, 2013; Kark et al., 2015; Hofmann et al., 2017). However, several problems remain to be addressed.

First, research exploring the relationship between the sense of control felt by higher education students and their safety behaviors during the COVID-19 pandemic remains largely lacking. Previous research indicated that individuals with sense of control engaged in violations of the norms (Miller and Mulligan, 2002; Leiter et al., 2009); however, Yu et al. (2021) believed that employees lacking of sense of control can increase their violations. The SARS-CoV-2 virus that causes COVID-19 is highly contagious, is often quite harmful, and has a long incubation period, resulting in uncertainty among the public (e.g., Dobson-Lohman and Potcovaru, 2020; Faasse and Newby, 2020; Lăzăroiu et al., 2020; Ljungholm and Olah, 2020; Maier and Brockmann, 2020). Therefore, in the context of the COVID-19 pandemic, the factors influencing the behavior of individuals are complex (Duncan, 2020; Sampson, 2020; Stevens, 2020), on higher education campuses in particular, with their dense populations and atmospheres of high uncertainty, students feel a lower level of control and show some differences compared to the general public in their safety behaviors for COVID-19 virus management. Therefore, the relationship between their sense of control and their safety behaviors in this context requires further research.

Second, previous studies have not fully elucidated the mechanism through which an individual’s sense of control influences their safety behavior. Drawing on psychological ownership theory, Liu et al. (2012) demonstrated that a greater sense of control can increase individuals’ sense of psychological ownership and motivate them to engage in the behaviors expected of them by their organization or leader (Liu et al., 2012). Meanwhile, drawing on the theory of planned behavior, Leiter et al. (2009) found that individuals with a sense of control overestimate their confidence when dealing with potential hazards and show risk-taking behavior. Further, studies drawing on conservation of resources theory (Hobfoll and Shirom, 2000; Hobfoll, 2001) have suggested that individuals’ behavioral patterns are influenced by the resources available to them. When resources (e.g., time, energy, cognitive attention, and willpower) are in abundance, individuals are more concerned with making a difference and tend to adopt facilitative behaviors to optimize their current environment and expand their resources. However, when resources are in short supply, individuals are more concerned with avoiding potential losses and display avoidant behaviors to maintain their current limited resources. Accordingly, higher education students with a higher sense of control may be expected to feel lower psychological stress and thus be strongly motivated to actively participate in safety management so as to expand their own resources (e.g., Halbesleben et al., 2014). Conversely, higher education students with a lower sense of control may be expected to feel higher psychological stress and thus comply with safety regulations in an effort to avoid potential loss of resources (e.g., Halbesleben et al., 2014; Jiménez et al., 2017). In light of the COVID-19 pandemic, there is an urgent need to further undertake research that explores the mechanism through which an individual’s sense of control influences their safety behavior.

Third, while previous studies have examined the contextual role of organizational or team culture on individual behavior in relation to “soft measures” (i.e., safety management measures that operate *via* encouragement or reward, such as the creation of a positive culture and provision of support or reward), they have not considered how harsh measures (i.e., safety management measures that operate *via* punishment, such as penalty, taunting, and the expression of negative emotions) and strict safety management systems can also magnify the positive effects of environmental factors (e.g., safety-specific transactional leadership) on individual safety behaviors (Smith et al., 2016). In the context of disease outbreak management, harsh measures (i.e., safety management measures by punishment, such as penalty, taunting, and expressing negative emotions) convey the message that participation in safety management is advocated by the epidemic control authorities and that violations of epidemic safety control policies will be severely punished (Huang et al., 2004). Such regulations provide guidance for individuals in their adoption of strategies to cope with the threats posed by the disease. Therefore, the situational role of harsh safety measures on the relationship between psychological stress and individual safety behavior needs to be explored.

**Figure 1:**
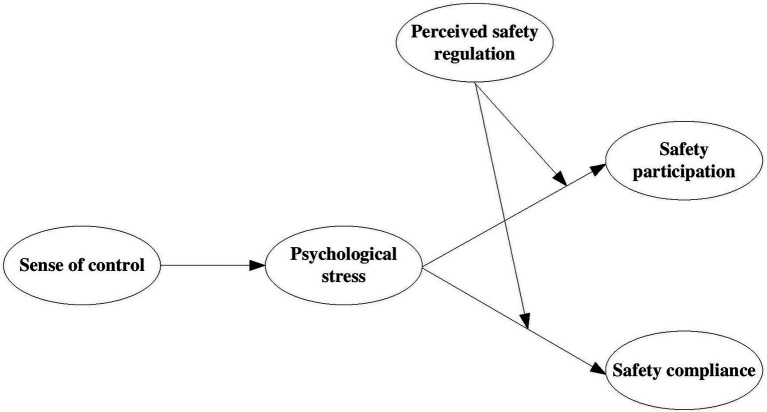
Hypothesized model.

Therefore, this study drew on conservation of resources theory to put forward and tests a mechanism through which individuals sense of control influences their safety compliance and participation in relation to COVID-19 prevention and control measures at a university. We proposed that psychological stress mediates the negative effect of sense of control on safety compliance and mediates the positive effect of sense of control on safety participation (see Figure 1). Further, this research tested the moderating effect of safety regulations and examined whether they served as a boundary condition to delimit the effect of students’ psychological stress on their safety compliance and participation (see Figure 1). Our results provide a benchmark against which efforts directed toward COVID-19 safety management in higher education can be assessed.

## Theoretical Background and Hypotheses

### The Conservation of Resources Theory

Conservation of resources theory (Halbesleben, 2006; Hobfoll, 2011) argues that individuals are motivated to protect their current resources and acquire potential resources. Previous research has suggested that when work resources (e.g., time, energy, cognitive attention, and willpower) are abundant, individuals focus on potential gains and think about how to improve their environment, thus adopting facilitative behaviors to expand their current work resources expand. Conversely, when work resources are scarce, individuals focus on potential losses and think about how to protect their resources from further depletion and loss, thus adopting avoidant behaviors to maintain their current work resources (Halbesleben, 2006). As this theory can describe the behavioral strategies that individuals take to actively handle their work resources under stress, it has been widely used to explain differences in individual behavioral patterns for relevant contexts (Grandey and Cropanzano, 1999 Brotheridge and Lee, 2002 Halbesleben, 2006).

We attempted to use conservation of resources theory to explain how and when sense of control influences safety behaviors among higher education students in the context of epidemic prevention and control in higher education institutions. The SARS-CoV-2 virus is highly contagious, has high infectivity, and has a long incubation period. Collectively, these properties create difficulties in detecting the spread of the virus, thus greatly disrupting the lives of teachers and students. While efforts are being undertaken to ensure effective prevention and control of COVID-19 in higher education institutions, students have to cope with not only their course schedules to meet their daily academic requirements, but also the risks associated with the spread of the virus and the protection of their own safety. Taken together, these burdens can lead to the rapid consumption of the students’ resources. In this scenario, students with a low sense of control may be expected to be more sensitive to losses such as being infected with COVID-19 due to their violation of safety policies and being punished for violating the regulations of their institution. They may tend to comply with the institution’s epidemic control system to preserve their limited resources and to avoid the negative consequences of infection.

In contrast, students with a high sense of control may be expected to be more sensitive to gains such as realizing the social value of self and winning respect from others. Such individuals may be inclined to participate in activities related to their institution’s epidemic management to improve the COVID-19 situation and facilitate their own resource acquisition.

### The Mediating Role of Psychological Stress

Having a sense of control can help higher education students alleviate their psychological stress. Sense of control refers to the belief that an individual has mastery over his or her life (Gurin et al., 1978; Kay et al., 2009). A lower sense of control means that individuals lose mastery of their environment, are more constrained by it, and thus have a higher level of life uncertainty and feel a higher level of psychological stress (Lachman and Weaver, 1998). Research has also indicated that individuals facing highly uncertain environments develop higher levels of psychological stress (Debus et al., 2012; De Berker et al., 2016; Lemée et al., 2019). During an epidemic, higher education students are not only faced with uncertainty regarding the future epidemic situation, but also struggle to respond effectively to the situation. Therefore, they experience a low level of control. In this scenario, students perceive a higher risk of viral infection and more serious infection hazards. They therefore have higher psychological stress. Previous research has indicated that individuals facing highly uncertain environments develop higher levels of psychological stress (Debus et al., 2012; De Berker et al., 2016; Lemée et al., 2019).

Psychological stress may reinforce individuals’ safety compliance. Psychological stress is defined as an unfavorable person-environment relationship (Lazarus, 1993), in which the highly stressed individual seeks to adapt to the environment to achieve a more favorable person-environment relationship, such as by following rules. Conservation of resources theory suggests that higher education students who experience higher psychological stress during the epidemic prevention and control may struggle to effectively and simultaneously cope with the risks posed by the COVID-19 virus and the requirements of their study tasks. To avoid facing the adverse effects of the external environment’s adverse effects on themselves, they may tend to comply with the institution’s epidemic control system. For example, they enter or leave the school only according to the school’s requirements, will wear masks according to the institution’s regulations, wear masks, and report their body temperature daily and punctually. Indeed, the results of previous studies indicate that they are likely to adopt risk-averse behaviors, such as compliance with safety regulations (Kark et al., 2015; Xia et al., 2017), to avoid unfavorable outcomes (Cooper et al., 2012). Thus, we proposed the following hypothesis:

*H1a*: Psychological stress mediates the relationship between higher education students’ sense of control and their safety compliance.

Psychological stress can also reduce individuals’ adoption of safety participation behaviors (e.g., Wang et al., 2018). Karabay (2014) found that, when individuals experience high levels of psychological stress, they focus more on their own work and engage in fewer organizational citizenship behaviors due to their limited resources. Conservation of resources theory suggests that higher education students with lower levels of psychological stress during an epidemic are better positioned for potential gains. Such individuals may tend to actively participate in epidemic safety management to enable effective control over the epidemic, reduce risks to the public.

Some previous studies also suggest that individuals with a stronger sense of control tend to engage in approach behavior (Keltner et al., 2003; Xu et al., 2020), such as participating in epidemic safety management. Thus, we proposed the following hypothesis:

*H1b*: Psychological stress mediates the relationship between higher education students’ sense of control and their safety participation.

### The Moderating Role of Safety Regulations

Strong safety regulations can reinforce the positive relationship between the psychological stress of higher education students and their safety compliance. The strength of safety regulations reflects the importance of implementing the safety policy and the monitoring process associated with the safety procedures (Huang et al., 2004). Safety regulations are an important indicator of a group’s safety practices (Zohar, 2000; Neal and Griffin, 2006). Stronger safety regulations indicate to group members that “ignoring safety policies can lead to serious consequences,” and that safety hazards can escalate into safety incidents and cause individuals to be severely punished. The risk of punishment drives members to examine their own safety behaviors and consider the likelihood and severity of consequent adverse outcomes.

In the context of COVID-19 prevention and control in higher education institutions, stronger safety regulations indicate to students that failure to comply with regulations may lead to serious epidemic risks and may cause the student to be punished. Hence, students treat safety compliance as a way to maintain the status quo and avoid possible losses, and tend to adhere to the institution’s epidemic control measures—for example, by wearing masks at school, avoiding crowded parties, and not leaving the campus freely to contact the general public. On the contrary, weaker safety regulations indicate to students that responses to the epidemic that deviate from the regulation recommendations will not lead to adverse outcomes. For example, regardless of the psychological pressure faced by individuals, they will form the perception that they are not susceptible to the virus even if they do not wear masks. Huang et al. (2004) showed when safety regulations are weak, safety violations will not lead to serious safety problems, which engenders a low level of safety compliance. Studies have also indicated that higher levels of safety regulations will enhance the role of environmental factors in shaping individual safety compliance (Probst, 2004; Clarke, 2006; Griffin and Hu, 2013). Therefore, we proposed the following hypothesis:

*H2a*: Safety regulations moderate the relationship between psychological stress and safety compliance, such that the relationship is more strongly positive when safety regulations are stronger, and vice versa.

Stronger safety regulations also indicate to students that participation in epidemic safety management is an effective way to control the spread of the virus and is valued by the institution. In this situation, under conditions of psychological stress, students treat participation in safety measures as an important way to realize the social value of self and actively engage in epidemic safety management to expand their resources. For example, they may express positive views of the epidemic safety control measures to other students and encourage them to comply.

Conversely, weaker safety regulations indicate to students that participation is not a means of improving public health. In this situation, under conditions of psychological stress, then, college students realize that participation in epidemic safety management does not help them gain personal resources, so they do not actively participate in carrying out. Studies have indicated that placing a priority on safety can influence the effects of environmental factors on safety participation (Clarke and Ward, 2006) and that a culture that prioritizes safety can buffer the negative effects of locus of control on safety participation (Cigularov et al., 2009). Thus, we proposed the following hypothesis:

*H2b*: Safety regulations moderate the relationship between psychological stress and safety participation, such that the relationship is more strongly negative when safety regulations are weaker, and vice versa.

## Materials and Methods

### Sample and Data Collection

More than 492 college students from 58 classes in a mainland China university participated by completing two surveys. To reduce common method bias, we used a two-wave lagged design with 2 weeks in between each data collection stage. In the first-round survey, college students reported on their sense of control, their psychological stress, and the perceived safety regulations. Two weeks later, the same students reported on their safety compliance with and participation in safety practices. In the first round of data collection, 490 college students completed the questionnaire; in the second round, 485 completed the questionnaire. After matching the responses, we were left with 481 valid questionnaires. The final sample included 264 males (54.9%) and 217 females (45.1%), of whom 33.7% were freshmen, 38% were sophomores, 25.8% were juniors, and 2.5% were seniors.

### Measurement

All of the scales used in this research were translated into Chinese using a rigorous back-translation process (Brislin, 1980). Specifically, we set up a research group consisting of two safety management researchers, two PhD in English candidates and three undergraduate students. We then translated the scales into Chinese and ensured that the undergraduate students fully understood the measurement questions. The two PhD in English candidates then back-translated the Chinese scales into English and compared the back-translated scales with the originals to ensure precision of meaning. Additionally, drawing on Kark et al. (2015), we specifically emphasized the context of COVID-19 in our scales (i.e., we added an introductory phrase indicating the epidemic management context, such as “During the COVID-19 pandemic,” to each measurement entry). To reduce common method bias, the variables were measured on a five-point Likert scale (1 = strongly disagree; 5 = strongly agree), except for safety regulations, which was measured on a seven-point Likert scale (1 = strongly disagree; 7 = strongly agree).

#### Sense of Control

Three items developed by Lachman and Weaver (1998) were used to measure sense of control. This scale contains items such as “[During the COVID-19 pandemic,] there is little I can do to change many of the important things in my life” (*α* = 0.82).

#### Psychological Stress

The four items from Motowidlo et al. (1986) were used to measure of psychological stress. A sample item is “I feel a great deal of stress because of COVID-19” (*α* = 0.92).

#### Safety Compliance

We adapted scale of Neal and Griffin (2006) to measure safety compliance in the context of the COVID-19 pandemic. For example, the original item “I use all the necessary safety equipment to do my job” was changed to “[During the COVID-19 pandemic, to prevent and control the spread of COVID-19] I use all the necessary safety equipment in my daily life” (*α* = 0.86).

#### Safety Participation

We adapted scale of Neal and Griffin (2006) to measure safety participation in the context of the COVID-19 pandemic. For example, the original item “I promote the safety program within the organization” was changed to “[During the COVID-19 pandemic, to prevent and control the spread of COVID-19] I promote the epidemic safety program within our university” (*α* = 0.85).

#### Perceived Safety Regulations

We adapted three items from Huang et al. (2004) to measure perceived safety regulations in the context of the COVID-19 pandemic. The original item “Employees always receive disciplinary action for a safety rule violation” was changed to “[During the COVID-19 pandemic] students always receive disciplinary action for violating a safety rules that prevent the spread of COVID-19.” The other two items are “During the COVID-19 pandemic, there has been a Safety Control Department in our university that works toward creating a safer work environment to prevent the spread of COVID-19 in the university” and “During the COVID-19 pandemic, students should be disciplined for violating safety rules that prevent the spread of COVID-19” (*α* = 0.96).

#### Control Variables

Drawing on previous research (Neal and Griffin, 2006; Kark et al., 2015), we controlled for individual demographic factors including grade level and gender. Drawing on finding of Kark et al. (2015) that an individual’s regulatory focus has a significant impact on safety behavior, we also controlled for an individual regulatory focus. Specifically, nine items were used each to measure individual promotion focus (Lockwood et al., 2002; *α* = 0.95) and prevention focus (Lockwood et al., 2002; *α* = 0.94) on a five-point Likert scale (1 = strongly disagree; 5 = strongly agree).

### Data Analysis

We first used Mplus 7.0 (Muthén and Muthén, 2012) to test the indirect effect of sense of control on safety compliance *via* psychological stress and the indirect effect of sense of control on safety participation *via* psychological stress. Then, also using Mplus 7.0, we tested the cross-level moderating effects of perceived safety regulations. Lastly, drawing on Dawson (2014), we plotted the moderating effects of perceived safety regulations.

## Results

Taking into consideration our sample size, we carried out item parceling by randomly creating three parcel items for constructs of more than three items (e.g., Little et al., 2002; DeRue and Wellman, 2009). The confirmatory factor analyses indicated that the seven-factor model (sense of control, psychological stress, safety compliance, safety participation, perceived safety regulation, promotion focus, and prevention focus) fits the data well (*χ*^2^ = 209.18, *df* = 168, CFI = 0.99, TLI = 0.99, RMSEA = 0.02, and SRMR = 0.03) and significantly better than the other models (See Table 1). The results from Harman’s one-factor test (Podsakoff et al., 2003) showed that no single factor accounted for the majority of the covariance among the latent factors (less than 20.9%), which indicated that common method bias did not have a substantial impact on this study. Descriptive analyses of the variables are shown in Table 2. Individual sense of control was significantly negatively related to psychological stress (*γ* = −0.39, *p* < 0.01), safety compliance (*γ* = −0.18, *p* < 0.01), and positively related to safety participation (*γ* = 0.17, *p* < 0.01), while psychological stress was significantly positively related to safety compliance (*γ* = 0.33, *p* < 0.01) and negatively related to safety participation (*γ* = −0.36, *p* < 0.01).

**Table 1:** Confirmatory factor analysis for testing structure validity.

Model	*χ* ^2^	*df*	*χ*^2^/df	CFI	TLI	RMSEA	SRMR
Seven factors (baseline model): sense of control, psychological stress, safety compliance, safety participation, perceived safety regulation, promotion focus, and prevention focus	209.18	168	1.25	0.99	0.99	0.02	0.03
Six factors: collapsing promotion focus and prevention focus	1679.58	174	9.65	0.81	0.77	0.13	0.11
Five factors: collapsing promotion focus and prevention focus, collapsing safety compliance and participation	2619.78	179	14.63	0.69	0.64	0.19	0.14
Four factors: collapsing promotion focus, prevention focus and perceived safety regulation, and collapsing safety compliance and safety participation	4062.04	183	22.20	0.51	0.44	0.21	0.17
Three factors: collapsing promotion focus, prevention focus, perceived safety regulation, safety compliance, and safety participation	4737.31	186	25.47	0.43	0.35	0.23	0.19
Two factors: collapsing promotion focus, prevention focus, perceived safety regulation, safety compliance, safety participation, and psychological stress	6198.78	188	32.97	0.24	0.15	0.26	0.19
One factor: collapsing all the variables	6601.38	189	34.93	0.19	0.10	0.26	0.19

**Table 2:** Descriptive statistics and variables correlation.

Variables	*M*	*SD*	1	2	3	4	5	6	7	8	9
1. Grade	1.97	0.83									
2. Gender	0.55	0.50	−0.04								
3. Promotion focus	3.96	0.81	0.04	−0.04	(0.95)						
4. Prevention focus	3.41	0.93	−0.02	0.09	0.05	(0.94)					
5. Sense of control	2.96	0.65	0.00	0.03	0.22^**^	−0.21	(0.82)				
6. Psychological stress	3.06	0.80	−0.06	−0.05	−0.12^**^	0.11^**^	−0.39^**^	(0.92)			
7. Safety compliance	3.88	0.71	−0.03	0.04	0.02	0.11^*^	−0.18^**^	0.33^**^	(0.86)		
8. Safety participation	3.92	0.78	0.07	0.04	0.07	−0.15^**^	0.17^**^	−0.36^**^	−0.10^*^	(0.85)	
9. Safety regulation	5.33	1.27	−0.09^*^	−0.05	−0.05	0.06	−0.32^**^	0.13^**^	0.27^**^	0.04	(0.96)

**n* = 481; 1 = freshmen, 2 = sophomores, 3 = were juniors, and 4 = seniors. 0 = female, 1 = male. Cronbach’s alphas are in the parentheses on the diagonal*.

* *p* *< 0.05;*

** *p < 0.01, two-tailed*.

### Mediation Effect Test

Hypotheses 1a and 1b suggested that students’ sense of control negatively affects safety compliance *via* psychological stress and positively influences safety participation *via* psychological stress. After controlling for the students’ gender, grade, promotion focus, and prevention focus (See Table 3), we found that sense of control was negatively correlated with psychological stress (*β* = −0.47, *p* < 0.01), psychological stress was positively related to safety compliance (*β* = 27, *p* < 0.01), and psychological stress was negatively related to safety participation (*β* = −0.34, *p* < 0.01). The results from our 20,000-sample bootstrapping analysis indicated that the indirect path from sense of control to safety compliance through psychological stress was significant (−0.13, 95% CI [−0.174, −0.078]). Therefore, Hypothesis 1a was supported. Meanwhile, the path from sense of control to safety participation through psychological stress was also significant (0.21, 95% CI [0.097, 0.220]). Thus, Hypothesis 1b was also supported.

**Table 3:** Meditation effect analysis.

Variables	Model 1		Model 2	Model 3	
	Safety compliance	Safety participation	Psychological stress	Safety compliance	Safety participation
Direct effect					
Gender	0.04 (0.06)	0.06 (0.07)	−0.08 (0.06)	0.06 (0.06)	0.04 (0.07)
Grade	−0.02 (0.04)	0.06 (0.04)	−0.06 (0.04)	−0.01 (0.03)	0.04 (0.04)
Promotion focus	0.06 (0.04)	−0.10^**^ (0.04)	0.03 (0.04)	0.05 (0.04)	−0.09^**^ (0.04)
Prevention focus	0.12^**^ (0.04)	0.14^**^ (0.03)	0.02 (0.05)	0.12^**^ (0.03)	0.15^**^ (0.04)
Sense of control	−0.19^**^ (0.06)	0.17^**^ (0.06)	−0.47^**^ (0.06)	−0.06 (0.06)	0.01 (0.06)
Psychological stress				0.27^**^ (0.04)	−0.34^**^ (0.06)
Indirect effect			95% CI, 20, 000 repetitions		
Sense of control → psychological stress → safety compliance				−0.13 [−0.174, −0.078]	
Sense of control → psychological stress → safety participation				0.21 [0.097, 0.220]	

*n = 481*.

*CI, confidence interval*.

** *p < 0.01*.

**Figure 2:**
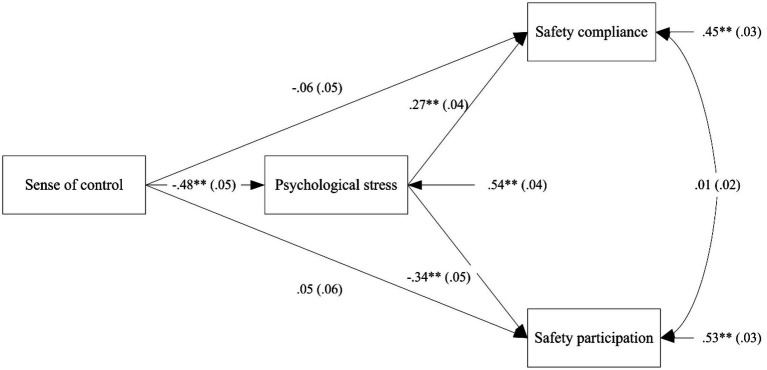
Path analysis of research model. **p < 0.01, two-tailed.

To more clearly represent the results of our data analysis, we conducted a full-model path analysis (Figure 2). The results showed that the indirect effect of “sense of control → psychological stress → safety compliance” was significant, while the indirect effect of “sense of control → psychological stress → safety participation” was also significant. These results suggest a divergent indirect path through which sense of control has an effect on individuals’ safety behavior.

### Moderation Effect Test

Hypotheses 2a suggested that perceived safety regulations moderate the relationship between students’ psychological stress and safety compliance.^1^ We found that when perceived safety regulations were high (+1 *SD*), the effect of students’ psychological stress on safety compliance was positive and significant (0.39, 95% CI [0.303, 0.483]; See Table 4). When perceived safety regulations were low (−1 *SD*), the effect of psychological stress on safety compliance was also positive and significant (0.14, 95% CI [0.006, 0.278]). Further, there was a significant difference in the indirect effect of individual psychological stress on safety compliance when perceived safety regulations were high vs. low (0.25, 95% CI [0.093, 0.409]). Therefore, Hypothesis 2a was supported.

**Table 4:** Moderation effect analysis.

Dependent variable	Moderator perceived safety regulation	Effect (*P_Y1M_*)	95% CI
Safety compliance	Low (−1 *SD*)	0.14^*^ (0.07)	[0.006, 0.278]
	High (+1 *SD*)	0.39^**^ (0.05)	[0.303, 0.483]
	Diff	0.25^**^ (0.08)	[0.093, 0.409]
**Dependent variable**	**Moderator perceived safety regulation**	**Effect (*P_Y2M_*)**	**95% CI**
Safety participation	Low (−1 *SD*)	−0.52^*^ (0.06)	[−0.646, −0.399]
	High (+1 *SD*)	−0.17^**^ (0.08)	[−0.317, −0.015]
	Diff	0.36^**^ (0.09)	[0.185, 0.528]

*n = 481*.

* *p* *< 0.05;*

** *p < 0.01*.

*P_Y1M_ refers to the effect of safety compliance on psychological stress, P_Y2M_ refers to the effect of safety participation on psychological stress; and Diff refers to the mediation effect difference between a high level and a low level of perceived safety regulation. CI, confidence interval*.

Hypotheses 2b suggested that perceived safety regulations moderate the relationship between students’ psychological stress and safety participation. The results showed that the effect of students’ psychological stress on safety participation was negative and significant when perceived safety regulations were high (+1 *SD*, −0.17, 95% CI [−0.317, −0.015]). This effect was also negative and significant when perceived safety regulations were low (−1 *SD*, −0.52, 95% CI [−0.646, −0.399]). Further, there was a significant difference in the effect of individual psychological stress on safety participation when perceived safety regulations were high vs. low (0.36, 95% CI [0.185, 0.528]). Therefore, Hypothesis 2b was supported.

**Figure 3:**
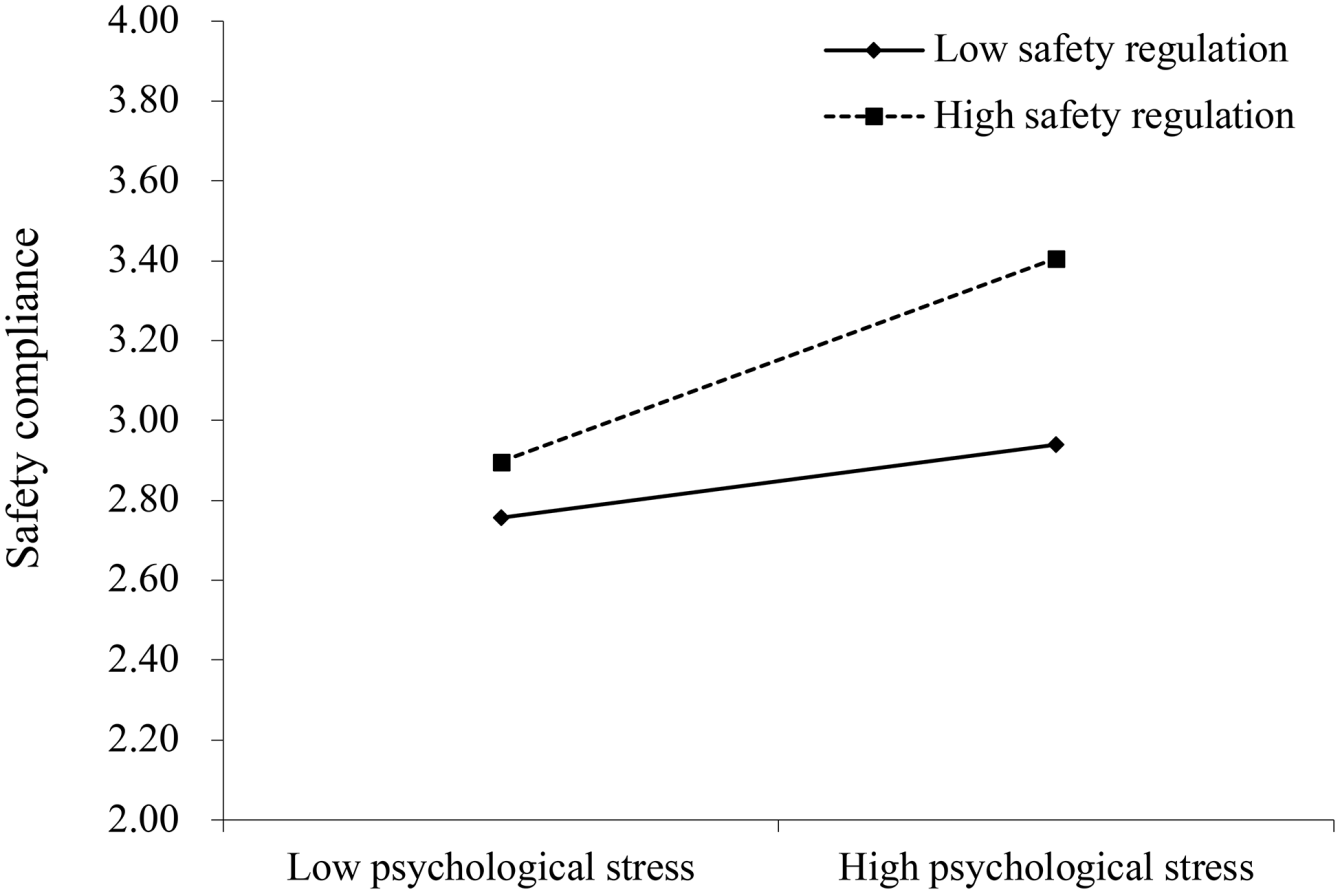
Moderation effect of perceived safety regulation on relationship between psychological stress and safety compliance.

Following Dawson (2014), we plotted the moderating effect of perceived safety regulations on the relationship between students’ psychological stress and safety compliance to visualize this effect (see Figure 3). Compared to the effect under perceived low safety regulations, the positive relationship between students’ psychological stress and safety compliance was magnified under the condition of perceived high safety regulations. This suggests that stronger safety regulations convey the message that students may be severely punished for violating rules aimed at preventing and controlling COVID-19 spread, thus inclining students toward complying with those rules to avoid possible punishment and losses. In contrast, the positive relationship between psychological stress and safety compliance was buffered, which suggests that weaker safety regulations convey the message that violating the rules will not lead to adverse results. In this case, students may turn a blind eye to safety regulations for the prevention and control of COVID-19 spread and demonstrate a low level of safety compliance.

**Figure 4:**
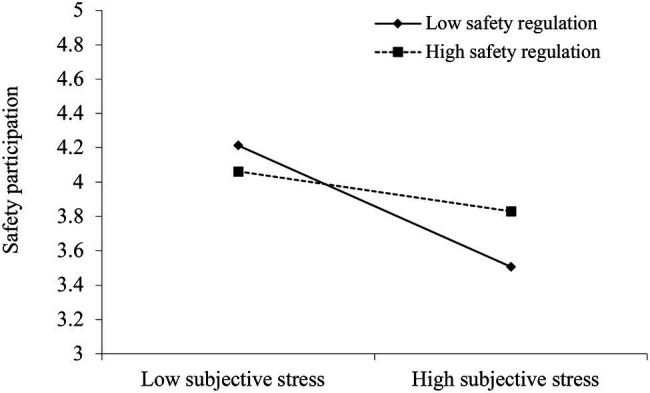
Moderation effect of perceived safety regulation on relationship between psychological stress on safety participation.

We also plotted the moderating effect of perceived safety regulations on the relationship between students’ psychological stress and safety participation (see Figure 4). The results suggest that stronger safety regulations signal the valuation of participation in epidemic safety management by the university. Therefore, students tend to treat safety participation as a way to realize social value of self, which prompts them to participate in epidemic safety management. In comparison, weaker safety regulations convey the message that participating in epidemic safety management has nothing to do with the prevention and control of COVID-19 spread. In this case, students may be careless about their safety and engage in safety measures only to a limited extent.

## Discussion

Drawing on conservation of resources theory, this study proposed and tested the mechanism underlying and boundary conditions delimiting the effect of students’ sense of control on safety compliance and safety participation in relation to the prevention and control of COVID-19 spread. The results showed that students’ perceived psychological stress mediated the negative effect of sense of control on safety compliance, as well as the positive effect of sense of control on safety participation. Further, perceived safety regulations reinforced the positive relationship between psychological stress and safety compliance, and buffered the negative relationship between psychological stress and safety participation.

### Theoretical Contributions

Our research makes several key theoretical contributions.

First, it deepens our understanding of the mechanisms underlying the effects of sense of control on safety behavior using conservation of resources theory. A previous study focusing on a motivational perspective found that a lower sense of control triggers individuals to focus on their short-term benefits and prompts them to engage in avoidance behaviors (Mittal and Griskevicius, 2014). Drawing on conservation of resources theory, this study adds to that finding by showing that in the context of COVID-19 safety management, students who perceive a lower sense of control face higher psychological stress and tend to engage in avoidant behaviors to preserve their limited resources, thereby exhibiting a higher level of safety compliance. Meanwhile, students who perceive a higher sense of control face less psychological stress and tend to expand their resources, engage in activities to realize their social values, and exhibit higher levels of safety participation. In this way, our study explains the divergent effect of sense of control on safety behaviors with psychological stress as the mediator. It therefore enriches our understanding of the effects of sense of control in a safety management context.

Second, by introducing the concept of perceived safety regulations, this study verifies the boundary conditions under which individuals’ psychological stress influences their compliance with regulations and their participation in regulation implementation. It therefore extends research on the contextual mechanisms underlying the relationship between psychological stress and safety performance. Previous studies have focused on the contextual effects of soft measures on the relationship between environmental factors, such as safety climate and individual safety behaviors (Zohar, 2000; Neal and Griffin, 2006; Xia et al., 2020). However, strict regulatory or punitive measures can also reinforce the influence of environmental factors on individuals’ safety behaviors (Huang et al., 2004). Focusing on harsh measures, this research indicates that stronger safety regulations signal to students that failure to comply with safety regulations for the prevention and control of COVID-19 spread may result in serious safety risks and severe punishment for violators, thus strengthening the positive relationship between psychological stress and safety compliance. In addition, stronger safety regulations convey the message that participation in activities for the prevention and control of COVID-19 spread is valued by higher education institutions and is an important way for students to realize their social values, thus buffering the negative relationship between psychological stress and safety participation. Therefore, by focusing on harsh measures in safety management, this study deepens our understanding of the contextual effects of safety regulations that influence the impact of individual psychological stress on safety behavior.

Third, by introducing conservation of resources theory to the context of epidemic control in higher education institutions, this study contributes to conservation of resources theory. It adds to previous research that has used this theory to explain the process by which environmental conditions affect individual safety behavior through resource depletion in safety management situations (Bacharach et al., 2008 Hammer et al., 2016; Kelloway, 2017). While efforts are being undertaken to prevent and control the spread of COVID-19 in higher education institutions, students have to cope with not only their course schedules to meet their daily academic requirements, but also the risks of virus infection and the protection of their own safety. In this situation, students’ resource consumption is more pronounced, while their safety behavioral patterns also vary considerably due to the different resources in their possession. This study differs from previous studies by focusing on the context of prevention and control during the COVID-19 pandemic and thus expands the range of scenarios to which conservation of resources theory has been applied.

### Practical Implications

We expect our study to have practical implications for the prevention and control of COVID-19 spread in higher education institutions. First, we found that a greater sense of control negatively affects safety compliance *via* psychological stress but positively impacts safety participation *via* psychological stress. Therefore, epidemic prevention and control departments in higher education institutions should be aware of the divergent effects of the sense of control on safety behavior. Due to their lack of knowledge about COVID-19, students may generally have a lower sense of control and show a higher level of safety compliance. However, due to concerns about their own health and the desire to avoid risks, students may tend to decline to participate in safety management activities. Therefore, managers could encourage students to actively participate in safety management by emphasizing that cooperation produces the best result, or by awarding and recognizing the residence or class that engages in safety participation. Support comes from research that has shown that shared goals promote individuals’ proactive behavior in safety management (e.g., Neal and Griffin, 2006).

Second, we found that perceived safety regulations reinforce the positive effect of psychological stress on safety compliance and buffer the negative effect of psychological stress on safety participation. Thus, managers in higher education institutions would be well advised to increase their efforts in implementing safety regulations, to enhance students’ compliance with safety regulations for the prevention and control of virus spread and to encourage them to fully engage in pandemic-related safety activities. For example, managers could develop stronger publicity campaigns to make students aware of the consequences of violating prevention and control policies. Managers could also award prizes to students who comply with safety regulations and actively participate in safety management activities, and punish those who break the rules.

### Limitations and Future Research

Like any research, this research has limitations. First, the sample for this study was obtained from a single university. Moreover, the differences in perceived safety regulations among the members of different classes were relatively small, so the data were not suitable for cross-level analysis. Therefore, it was difficult to parse out the cross-level moderating effect of perceived safety regulations on the relationship between sense of control and safety compliance. Chowdhury and Endres (2010) concluded that cross-level analyses are effective for distinguishing between-group from within-group effects and for observing the effects of high-level variables on multiple dimensions of individual behavioral patterns. Thus, future research could expand the range of sample source and use cross-level analyses to explore the contextual effects of group safety regulations on the relationship between students’ sense of control and their compliance.

Second, all of the variables in our study were measured by self-report, which may have led to the results being influenced by the participants’ sense of social desirability (Crowne and Marlowe, 1960). Thus, future research could use other-rated measures of individual safety compliance or participation. For example, researchers could ask teachers or administrators to rate students’ safety compliance and participation, or use objective indicators to measure individual behavior, such as the number of violations or points for participation in safety management activities. This study also used a survey research method, which could not verify the causal relationship between the variables. Future research could use an experimental approach to verify the causal relationship between sense of control and safety behavior. Specifically, researchers could prime different levels of sense of control in different groups, then observe whether there were significant differences in safety compliance and safety participation between individuals in the experimental group and control groups.

Third, several alternative mechanisms might potentially explain the relationship between individuals’ sense of control and their safety behavior. For example, according to the theory of planned behavior (Ajzen, 1991), individuals with a higher sense of control believe that they are in control of the external environment and thus show a greater tendency to comply with and participate in safety activities. Also, according to social learning theory (Bandura, 1977), individuals with a low sense of control are more sensitive to losses and tend to imitate the behavior of others in an effort to reduce the impact of uncertainty; thus, they may be expected to willingly comply with safety measures to prevent and control the spread of COVID-19. Thus, future researchers could draw on these alternative theories to explore other mechanisms underlying and situational conditions determining the relationship between students’ sense of control and their safety compliance and participation.

## Conclusion

By focusing on safety management of the COVID-19 pandemic at a university, this study examined how and when sense of control affects student’s safety compliance and safety participation. Based on two-wave time-lagged data collected from 481 students in 58 classes, our findings showed that students’ psychological stress mediated the negative effect of sense of control on safety compliance and mediated the positive effect of sense of power on safety participation. Moreover, we found that perceived safety regulations strengthened the positive relationship between students’ psychological stress and safety compliance, and buffered the negative effects of psychological stress on safety participation. Our research advances the understanding of the underlying psychological mechanisms through which sense of control affects safety behavior in the context of COVID-19 pandemic management in higher education institutions, and of how perceived safety regulations affect this psychological process.

## Data Availability Statement

The raw data supporting the conclusions of this article will be made available by the authors, without undue reservation.

## Ethics Statement

This study was reviewed and approved by Academic Committee of Zhejiang University. Written informed consent was obtained from all participants for their participation in this study, in accordance with the Declaration of Helsinki.

## Author Contributions

PL mainly proposed the initial idea and basic model for this research, wrote the introduction part, and contributed to collect and analyze the data of this study. HZ mainly provided his knowledge to perfect the research model and develop the theatrical hypotheses. All authors contributed to the article and approved the submitted version.

## Funding

This paper is supported by the Humanities and Social Sciences Youth Fund Project from Ministry of Education under Grant Nos. 17YJC880020 and 18YJC880114.

## Conflict of Interest

The authors declare that the research was conducted in the absence of any commercial or financial relationships that could be construed as a potential conflict of interest.

## Publisher’s Note

All claims expressed in this article are solely those of the authors and do not necessarily represent those of their affiliated organizations, or those of the publisher, the editors and the reviewers. Any product that may be evaluated in this article, or claim that may be made by its manufacturer, is not guaranteed or endorsed by the publisher.

## Footnotes

^1^The results did not change significantly when we added the control variables, so we present the results with the focal variable only (i.e., including sense of control, psychological stress, safety compliance, safety participation, and perceived safety regulations).

## References

[ref1] Ajzen I. (1991). The theory of planned behavior. Organ. Behav. Hum. Decis. Process. 50, 179–211. doi: 10.1016/0749-5978(91)90020-T

[ref006] Bacharach S. B. Bamberger P. A. Doveh E. (2008). Firefighters, critical incidents, and drinking to cope: The adequacy of unit-level performance resources as a source of vulnerability and protection. J. Appl. Psychol. 93, 155–169. doi: 10.1037/0021-9010.93.1.155 18211142

[ref2] Bandura A. (1977). Social Learning Theory. Englewood Cliffs, NJ: Prentice-Hall.

[ref3] Brislin R. W. (1980). “Translation and content analysis of oral and written material,” in Handbook of Crosscultural Psychology. *Vol*. 2. eds. Triandis H. C. Berry J. W. (Boston, MA: Allyn & Bacon), 389–444.

[ref003] Brotheridge C. M. Lee R. T. (2002). Testing a conservation of resources model of the dynamics of emotional labor. J. Occup. Health Psychol. 7, 57–67. doi: 10.1037/1076-8998.7.1.57, PMID: 11827234

[ref4] Cheng S. Y. Wang C. J. Shen A. C. T. Chang S. C. (2020). How to safely reopen colleges and universities during COVID-19: experiences from Taiwan. Ann. Intern. Med. 173, 638–641. doi: 10.7326/M20-2927, PMID: 32614638 PMC7339040

[ref5] Chowdhury S. K. Endres M. L. (2010). The impact of client variability on nurses’ occupational strain and injury: cross-level moderation by safety climate. Acad. Manag. J. 53, 182–198. doi: 10.5465/amj.2010.48037720

[ref6] Cigularov K. P. Chen P. Y. Stallones L. (2009). Error communication in young farm workers: its relationship to safety climate and safety locus of control. Work Stress. 23, 297–312. doi: 10.1080/02678370903416679

[ref7] Clarke S. (2006). The relationship between safety climate and safety performance: a meta-analytic review. J. Occup. Health Psychol. 11, 315–327. doi: 10.1037/1076-8998.11.4.315, PMID: 17059296

[ref8] Clarke S. Ward K. (2006). The role of leader influence tactics and safety climate in engaging employees’ safety participation. Risk Anal. 26, 1175–1185. doi: 10.1111/j.1539-6924.2006.00824.x, PMID: 17054524

[ref9] Cooper T. Faseruk A. Smith J. (2012). A review of BASEL III–research questions and potential impacts. GSTE Bus. Rev. 1, 18–23.

[ref10] Crowne D. P. Marlowe D. (1960). A new scale of social desirability independent of psychopathology. J. Consult. Psychol. 24, 349–354. doi: 10.1037/h0047358, PMID: 13813058

[ref004] Dawson J. F. (2014). Moderation in Management Research: What, Why, When, and How. J. Bus. Psychol. 29, 1–9. doi: 10.1007/s10869-013-9308-7

[ref11] De Berker A. O. Rutledge R. B. Mathys C. Marshall L. Cross G. F. Dolan R. J.. (2016). Computations of uncertainty mediate acute stress responses in humans. Nat. Commun. 7:10996. doi: 10.1038/ncomms10996, PMID: 27020312 PMC4820542

[ref12] Debus M. E. Probst T. M. König C. J. Kleinmann M. (2012). Catch me if I fall! Enacted uncertainty avoidance and the social safety net as country-level moderators in the job insecurity–job attitudes link. J. Appl. Psychol. 97, 690–698. doi: 10.1037/a0027832, PMID: 22448808

[ref13] DeRue D. S. Wellman N. (2009). Developing leaders via experience: the role of developmental challenge, learning orientation, and feedback availability. J. Appl. Psychol. 94, 859–875. doi: 10.1037/a0015317, PMID: 19594230

[ref14] Dobson-Lohman E. Potcovaru A. M. (2020). Fake news content shaping the COVID-19 pandemic fear: virus anxiety, emotional contagion, and responsible media reporting. Anal. Metaphys. 19, 94–100. doi: 10.22381/AM19202011

[ref15] Duncan C. (2020). Gender-related depression, anxiety, and psychological stress experienced during the COVID-19 pandemic. J. Res. Gend. Stud. 10, 84–94. doi: 10.22381/JRGS10220204

[ref16] Faasse K. Newby J. (2020). Public perceptions of COVID-19 in Australia: perceived risk, knowledge, health-protective behaviors, and vaccine intentions. Front. Psychol. 11:551004. doi: 10.3389/fpsyg.2020.551004, PMID: 33117223 PMC7561403

[ref002] Grandey A. A. Cropanzano R. (1999). The conservation of resources model applied to work–family conflict and strain. J. Vocat. Behav. 54, 350–370. doi: 10.1006/jvbe.1998.1666

[ref17] Griffin M. A. Hu X. (2013). How leaders differentially motivate safety compliance and safety participation: the role of monitoring, inspiring, and learning. Saf. Sci. 60, 196–202. doi: 10.1016/j.ssci.2013.07.019

[ref18] Griffin M. A. Neal A. (2000). Perceptions of safety at work: a framework for linking safety climate to safety performance, knowledge, and motivation. J. Occup. Health Psychol. 5, 347–358. doi: 10.1037/1076-8998.5.3.347, PMID: 10912498

[ref19] Gurin P. Gurin G. Morrison B. M. (1978). Personal and ideological aspects of internal and external control. Soc. Psychol. 41, 275–296. doi: 10.2307/3033581

[ref001] Halbesleben J. R. B. (2006). Sources of social support and burnout: A meta-analytic test of the conservation of resources model. J. Appl. Psychol. 91, 1134–1145. doi: 10.1037/0021-9010.91.5.1134 16953774

[ref20] Halbesleben J. R. Neveu J. P. Paustian-Underdahl S. C. Westman M. (2014). Getting to the “COR” understanding the role of resources in conservation of resources theory. J. Manag. 40, 1334–1364. doi: 10.1177/0149206314527130

[ref21] Hammer L. B. Johnson R. C. Crain T. L. Bodner T. Kossek E. E. Davis K. D.. (2016). Intervention effects on safety compliance and citizenship behaviors: evidence from the work, family, and health study. J. Appl. Psychol. 101, 190–208. doi: 10.1037/apl0000047, PMID: 26348479 PMC4564872

[ref22] Hobfoll S. E. (2001). The influence of culture, community, and the nested-self in the stress process: advancing conservation of resources theory. Appl. Psychol. 50, 337–421. doi: 10.1111/1464-0597.00062

[ref008] Hobfoll S. E. (2011). Conservation of resource caravans and engaged settings. J. Occup. Organ. Psychol. 84, 116–122.

[ref23] Hobfoll S. E. Shirom A. (2000). “Conservation of resources theory: applications to stress and management in the workplace,” in Handbook of Organization Behavior. 2nd *Edn*. ed. Golembiewski R. T. (New York, NY: Dekker), 57–81.

[ref24] Hofmann D. A. Burke M. J. Zohar D. (2017). 100 years of occupational safety research: from basic protections and work analysis to a multilevel view of workplace safety and risk. J. Appl. Psychol. 102, 375–388. doi: 10.1037/apl0000114, PMID: 28125258

[ref25] Huang Y. H. Chen P. Y. Krauss A. D. Rogers D. A. (2004). Quality of the execution of corporate safety policies and employee safety outcomes: assessing the moderating role of supervisor safety support and the mediating role of employee safety control. J. Bus. Psychol. 18, 483–506. doi: 10.1023/B:JOBU.0000028448.01394.bf

[ref26] Jiménez M. G. Montorio I. Izal M. (2017). The association of age, sense of control, optimism, and self-esteem with emotional distress. Dev. Psychol. 53, 1398–1403. doi: 10.1037/dev0000341, PMID: 28459257

[ref27] Karabay M. E. (2014). An investigation of the effects of work-related stress and organizational commitment on organizational citizenship behavior: a research on banking industry. J. Bus. Res. 6, 282–302. doi: 10.20491/isader.2014115975

[ref28] Kark R. Katz-Navon T. Delegach M. (2015). The dual effects of leading for safety: the mediating role of employee regulatory focus. J. Appl. Psychol. 100, 1332–1348. doi: 10.1037/a0038818, PMID: 25664472

[ref29] Kay A. C. Whitson J. A. Gaucher D. Galinsky A. D. (2009). Compensatory control: achieving order through the mind, our institutions, and the heavens. Curr. Dir. Psychol. Sci. 18, 264–268. doi: 10.1111/j.1467-8721.2009.01649.x

[ref30] Keltner D. Gruenfeld D. H. Anderson C. (2003). Power, approach, and inhibition. Psychol. Rev. 110, 265–284. doi: 10.1037/0033-295X.110.2.265 12747524

[ref007] Kelloway E. K. (2017). Mental health in the workplace: Towards evidence-based practice. Canadian Psychology/Psychologie canadienne 58, 1–6. doi: 10.1037/cap0000084, PMID: 8434890

[ref32] Lachman M. E. Weaver S. L. (1998). The sense of control as a moderator of social class differences in health and well-being. J. Pers. Soc. Psychol. 74, 763–773. doi: 10.1037/0022-3514.74.3.763, PMID: 9523418

[ref33] Lăzăroiu G. Horak J. Valaskova K. (2020). Scaring ourselves to death in the time of COVID-19: pandemic awareness, virus anxiety, and contagious fear. Linguist. Philos. Invest. 19, 114–120. doi: 10.22381/LPI1920208

[ref34] Lazarus R. S. (1993). From psychological stress to the emotions: a history of changing outlooks. Annu. Rev. Psychol. 44, 1–22. doi: 10.1146/annurev.ps.44.020193.000245, PMID: 8434890

[ref35] Leiter M. P. Zanaletti W. Argentero P. (2009). Occupational risk perception, safety training, and injury prevention: testing a model in the Italian printing industry. J. Occup. Health Psychol. 14, 1–10. doi: 10.1037/1076-8998.14.1.1, PMID: 19210042

[ref36] Lemée C. Fleury-Bahi G. Navarro O. (2019). Impact of place identity, self-efficacy and anxiety state on the relationship between coastal flooding risk perception and the willingness to cope. Front. Psychol. 10:499. doi: 10.3389/fpsyg.2019.00499, PMID: 30915001 PMC6421279

[ref37] Little T. D. Cunningham W. A. Shahar G. Widaman K. F. (2002). To parcel or not to parcel: exploring the question, weighing the merits. Struct. Equ. Model. 9, 151–173. doi: 10.1207/S15328007SEM0902_1

[ref38] Liu J. Wang H. Hui C. Lee C. (2012). Psychological ownership: how having control matters. J. Manag. Stud. 49, 869–895. doi: 10.1111/j.1467-6486.2011.01028.x

[ref39] Ljungholm D. P. Olah M. L. (2020). Regulating fake news content during COVID-19 pandemic: evidence-based reality, trustworthy sources, and responsible media reporting. Rev. Contemp. Philos. 19, 43–49. doi: 10.22381/RCP1920203

[ref40] Lockwood P. Jordan C. H. Kunda Z. (2002). Motivation by positive or negative role models: regulatory focus determines who will best inspire us. J. Pers. Soc. Psychol. 83, 854–864. doi: 10.1037/0022-3514.83.4.854, PMID: 12374440

[ref41] Maier B. F. Brockmann D. (2020). Effective containment explains subexponential growth in recent confirmed COVID-19 cases in China. Science 368, 742–746. doi: 10.1126/science.abb4557, PMID: 32269067 PMC7164388

[ref42] Miller R. L. Mulligan R. D. (2002). Terror management: the effects of mortality salience and locus of control on risk-taking behaviors. Personal. Individ. Differ. 33, 1203–1214. doi: 10.1016/S0191-8869(02)00009-0

[ref43] Mittal C. Griskevicius V. (2014). Sense of control under uncertainty depends on people’s childhood environment: a life history theory approach. J. Pers. Soc. Psychol. 107, 621–637. doi: 10.1037/a0037398, PMID: 25133717

[ref44] Motowidlo S. J. Packard J. S. Manning M. R. (1986). Occupational stress: its causes and consequences for job performance. J. Appl. Psychol. 71, 618–629. doi: 10.1037/0021-9010.71.4.618 3804934

[ref45] Muthén L. K. Muthén B. O. (2012). Mplus Version 7 User’s Guide. Los Angeles, CA: Muthén & Muthén.

[ref46] Neal A. Griffin M. A. (2006). A study of the lagged relationships among safety climate, safety motivation, safety behavior, and accidents at the individual and group levels. J. Appl. Psychol. 91, 946–953. doi: 10.1037/0021-9010.91.4.946, PMID: 16834517

[ref005] Podsakoff P. M. MacKenzie S. B. Lee J.-Y. Podsakoff N. P. (2003). Common method biases in behavioral research: A critical review of the literature and recommended remedies. J. Appl. Psychol. 88, 879–903. doi: 10.1037/0021-9010.88.5.879 14516251

[ref47] Probst T. M. (2004). Safety and insecurity: exploring the moderating effect of organizational safety climate. J. Occup. Health Psychol. 9, 3–10. doi: 10.1037/1076-8998.9.1.3, PMID: 14700454

[ref49] Sampson G. (2020). Gender-based differences of contagious negative emotions, notable psychological distress, and mental health burden during the COVID-19 outbreak. J. Res. Gend. Stud. 10, 128–137. doi: 10.22381/JRGS10220208

[ref50] Smith T. D. Eldridge F. DeJoy D. M. (2016). Safety-specific transformational and passive leadership influences on firefighter safety climate perceptions and safety behavior outcomes. Saf. Sci. 86, 92–97. doi: 10.1016/j.ssci.2016.02.019

[ref51] Stevens A. (2020). Gender differences in COVID-19 sentiments, attitudes, habits, and behaviors: an empirical research. J. Res. Gend. Stud. 10, 95–105. doi: 10.22381/JRGS10220205

[ref52] Wang C. Cheng Z. Yue X. G. McAleer M. (2020). Risk management of Covid-19 by universities in China. J. Risk Fin. Manag. 13, 36–42. doi: 10.3390/jrfm13020036

[ref53] Wang D. Wang X. Xia N. (2018). How safety-related stress affects workers’ safety behavior: the moderating role of psychological capital. Saf. Sci. 103, 247–259. doi: 10.1016/j.ssci.2017.11.020

[ref54] Xia N. Wang X. Griffin M. A. Wu C. Liu B. (2017). Do we see how they perceive risk? An integrated analysis of risk perception and its effect on workplace safety behavior. Accid. Anal. Prev. 106, 234–242. doi: 10.1016/j.aap.2017.06.010, PMID: 28645020

[ref55] Xia N. Xie Q. Hu X. Wang X. Meng H. (2020). A dual perspective on risk perception and its effect on safety behavior: a moderated mediation model of safety motivation, and supervisor’s and coworkers’ safety climate. Accid. Anal. Prev. 134:105350. doi: 10.1016/j.aap.2019.105350, PMID: 31715549

[ref56] Xu Q. Kwan C. M. Zhou X. (2020). Helping yourself before helping others: how sense of control promotes charitable behaviors. J. Consum. Psychol. 30, 486–505. doi: 10.1002/jcpy.1163

[ref58] Yu X. Mehmood K. Paulsen N. Ma Z. Kwan H. K. (2021). Why safety knowledge cannot be transferred directly to expected safety outcomes in construction workers: the moderating effect of physiological perceived control and mediating effect of safety behavior. J. Constr. Eng. Manag. 147:04020152. doi: 10.1061/(ASCE)CO.1943-7862.0001965

[ref59] Zohar D. (2000). A group-level model of safety climate: testing the effect of group climate on microaccidents in manufacturing jobs. J. Appl. Psychol. 85, 587–596. doi: 10.1037/0021-9010.85.4.587, PMID: 10948803

